# Transcriptional, Functional, and Mechanistic Comparisons of Stem Cell–Derived Hepatocytes, HepaRG Cells, and Three-Dimensional Human Hepatocyte Spheroids as Predictive In Vitro Systems for Drug-Induced Liver Injury

**DOI:** 10.1124/dmd.116.074369

**Published:** 2017-04

**Authors:** Catherine C. Bell, Volker M. Lauschke, Sabine U. Vorrink, Henrik Palmgren, Rodger Duffin, Tommy B. Andersson, Magnus Ingelman-Sundberg

**Affiliations:** Section of Pharmacogenetics, Department of Physiology and Pharmacology, Karolinska Institutet, Stockholm, Sweden (C.C.B., V.M.L., S.U.V., T.B.A., M.I.-S.); Cardiovascular and Metabolic Diseases, Innovative Medicines and Early Development Biotech Unit, AstraZeneca, Mölndal, Sweden (H.P., T.B.A.); and CXR Biosciences Ltd., Dundee, United Kingdom (R.D.)

## Abstract

Reliable and versatile hepatic in vitro systems for the prediction of drug pharmacokinetics and toxicity are essential constituents of preclinical safety assessment pipelines for new medicines. Here, we compared three emerging cell systems—hepatocytes derived from induced pluripotent stem cells, HepaRG cells, and three-dimensional primary human hepatocyte (PHH) spheroids—at transcriptional and functional levels in a multicenter study to evaluate their potential as predictive models for drug-induced hepatotoxicity. Transcriptomic analyses revealed widespread gene expression differences between the three cell models, with 8148 of 17,462 analyzed genes (47%) being differentially expressed. Expression levels of genes involved in the metabolism of endogenous as well as xenobiotic compounds were significantly elevated in PHH spheroids, whereas genes involved in cell division and endocytosis were significantly upregulated in HepaRG cells and hepatocytes derived from induced pluripotent stem cells, respectively. Consequently, PHH spheroids were more sensitive to a panel of drugs with distinctly different toxicity mechanisms, an effect that was amplified by long-term exposure using repeated treatments. Importantly, toxicogenomic analyses revealed that transcriptomic changes in PHH spheroids were in compliance with cholestatic, carcinogenic, or steatogenic in vivo toxicity mechanisms at clinically relevant drug concentrations. Combined, the data reveal important phenotypic differences between the three cell systems and suggest that PHH spheroids can be used for functional investigations of drug-induced liver injury in vivo in humans.

## Introduction

Drug-induced liver injury (DILI) poses a serious threat to patients, accounting for 13% of acute liver failures and 15% of liver transplantations (Ostapowicz et al., 2002; Russo et al., 2004). Idiosyncratic DILI events, which are typically delayed in onset and restricted to predisposed individuals, account for 10% of these cases (Kaplowitz, 2005; Lauschke and Ingelman-Sundberg, 2016) and occur with an overall incidence of about 13–19 per 100,000 individuals (Sgro et al., 2002; Björnsson et al., 2013). Adverse drug reactions significantly increase the length and costs of hospitalization by 1.9 days and US$2262–3244, respectively, and are associated with a 1.9-fold increased mortality risk (Bates et al., 1997; Classen et al., 1997). Moreover, hepatic liabilities are important cost drivers for the pharmaceutical industry that can result in late-stage attrition of drug candidates or postmarketing withdrawals, as exemplified by bromfenac, troglitazone, ximelagatran, and pemoline (Park et al., 2011; Cook et al., 2014). In addition, decreased prescribing due to black box warnings reduces sales, and 10 of 45 compounds that were endowed with such boxed warnings between 1975 and 2000 received their label due to hepatotoxicity (Lasser et al., 2002).

Toxicity prediction of newly developed compounds in preclinical stages encompasses an array of in silico, in vitro, and in vivo studies. Animal testing has long been the cornerstone for safety assessments of novel chemical entities. Yet the liver is an organ with pronounced species differences with regard to expression and catalytic activities of factors involved in drug absorption, distribution, metabolism, and excretion (ADME). Therefore, animal models do not accurately replicate the etiology and pathogenesis of human liver injury. Thus, due to growing recognition of the limited predictive validity of animal models and increasing legislative pressure to reduce, refine, or replace (“3R” concept) the use of animal models, there is a clear need for predictive in vitro models, which faithfully reflect human liver physiology and function (Chapman et al., 2013).

Hepatic cell lines are frequently employed in preclinical screening assays, due to their ease of use, ready availability, and low costs. Importantly, however, most hepatic cell lines lack relevant hepatic phenotypes, due to limited expression of drug-metabolizing enzymes, which makes extrapolation of the results to humans questionable (Gerets et al., 2012). The HepaRG cell line presents a cell system that has been reported to be phenotypically stable, thus allowing long-term culture and repeated-exposure studies (Klein et al., 2014). Induced pluripotent stem cells (iPSCs) have the advantage that they can be generated from any human cell type, which allows the retrospective acquisition of cellular material from individuals with a particular genotype or phenotype of interest, such as an idiosyncratic adverse drug reaction, providing an interesting model for deciphering mechanisms of genetically determined DILI reactions (Kia et al., 2013).

Primary human hepatocytes (PHHs) are considered the gold standard for studying liver function (Gómez-Lechón et al., 2014). However, their rapid dedifferentiation in conventional two-dimensional (2D) monolayer cultures, paralleled by a loss of hepatic functionality, renders them unsuitable for long-term studies and significantly impairs their predictive power for DILI risk (Gerets et al., 2012; Lauschke et al., 2016c; Sison-Young et al., 2016; Heslop et al., 2017). To prevent dedifferentiation, an array of three-dimensional (3D) culture techniques has been developed in which hepatic phenotypes are maintained for extended periods of time (Lauschke et al., 2016a). One promising strategy is the culture of PHHs as 3D spheroidal aggregates in which hepatocyte-specific functions can be retained for several weeks (Bell et al., 2016), thus enabling repeated-exposure experiments.

In this study, we characterized the transcriptomic signatures of HepaRG cells, PHH spheroid cultures, and hepatocyte-like cells (HLCs) derived from iPSCs (hiPS-Hep cells). Whereas expression patterns in PHH spheroids resembled freshly isolated hepatocytes, HepaRG and hiPS-Hep cells exhibited widespread differences in gene expression, particularly in genes involved in the metabolism of endogenous and xenobiotic compounds. These gene expression differences translated into functional differences as assessed by the sensitivity toward six different hepatotoxic compounds, with PHH spheroids constituting the most sensitive model that detected hepatotoxicity at clinically relevant concentrations. Importantly, toxicogenomic analyses revealed that transcriptional responses elicited by compounds causing inhibition of mitochondrial respiration, perturbation of *β*-oxidation, cholestatic injury, or genotoxicity in vivo were faithfully reflected in this model. Combined, our data indicate that phenotypes and sensitivities to hepatotoxic agents differ considerably between preclinical cell models and that PHH spheroids are more physiologically relevant and mechanistically accurate in detecting and investigating hepatic liabilities of drugs as compared with HepaRG and hiPS-Hep cells.

## Materials and Methods

### 

#### Cell Culture.

Cryopreserved PHH 3D spheroids were cultured in culture medium (Williams’ E medium supplemented with 2 mM l-glutamine, 100 U/ml penicillin, 100 *µ*g/ml streptomycin, 10 *µ*g/ml insulin, 5.5 *µ*g/ml transferrin, 6.7 ng/ml sodium selenite, and 100 nM dexamethasone), as previously described (Bell et al., 2016). Four days after seeding, 50% of the culture medium was substituted with fresh fetal bovine serum (FBS)–free medium and the medium was subsequently exchanged daily until the start of treatment at day 7. Hepatocytes in monolayer culture were seeded into plates coated with 5 *μ*g/cm^2^ Rat Tail Collagen Type I (Corning, Corning, NY) in culture medium with 10% FBS. After 2 hours of attachment, the medium was replaced with serum-free culture medium. Donor demographics for all PHH used in this study are presented in Table 1. hiPS-Hep cells were obtained by differentiation from the human iPSC line ChiPSC18 (DEF-hiPSC ChiPSC18) (Cellartis; Takara Bio Europe AB, Göteborg, Sweden) using the Cellartis DE Differentiation Kit and the Cellartis HEP Differentiation Kit (Takara Bio Europe AB) according to the manufacturer’s instructions. After initiation of differentiation at day 22, the HLCs were dissociated and reseeded in an appropriate cell culture format for transcriptional analyses and viability assessments. HepaRG cells (Biopredic International, Saint Grégoire, France) were cultured and maintained in culture medium (Williams’ E basal medium plus GlutaMAX containing phenol red; Invitrogen, Carlsbad, CA) with Additive 710 (Biopredic International). For differentiation, cells were cultured in culture medium with Additive 720 (Biopredic International). Cells were maintained in growth medium for 2 weeks followed by 2 weeks of differentiation medium. The medium was changed to culture medium without phenol red and dimethylsulfoxide (DMSO) 1 day prior to the initiation of treatment.

**TABLE 1:** Demographic information of PHH donors used in this study

Donor	Sex	Age	Race	Viability of Isolated Cells
		*yr*		*%*
1	Male	22	Caucasian	83
2	Female	37	Asian	85
3	Male	58	Caucasian	79
4	Female	48	Polynesian	84

#### Compound Exposure and Generation of Toxicity Curves.

Compounds were dissolved in DMSO and diluted in FBS-free medium to a final DMSO concentration of 0.4%. Treatment was performed every 2 to 3 days in FBS-free medium. In the acute setting, viability was determined after a single-dose exposure for 2 days. Under long-term treatment, cells were repeatedly treated for 7 days (three exposures) and 14 days (six exposures). Viability, as assessed by cellular ATP levels, was determined using the CellTiter-Glo Luminescent Cell Viability Assay (Promega, Nacka, Sweden). Luminescence was measured and the samples were blank corrected and normalized to vehicle control. IC_50_ values were calculated using a sigmoidal dose-response regression model constrained at viability 0 and 100 (GraphPad Prism software; GraphPad Inc., La Jolla, CA). IC_10_ values were calculated as follows, with *x* = 10:


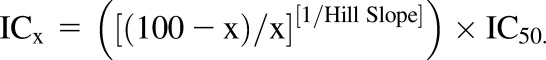


#### Transcriptomic Analyses.

After 2, 7, and 14 days in culture, cells were harvested in RNAprotect Cell Reagent (Qiagen, Sollentuna, Sweden). RNA was extracted with the AllPrep DNA/RNA Mini Kit according to the manufacturer’s instructions (Qiagen). Total RNA samples (45 ng) were labeled with cyanine 3, hybridized on Agilent Whole Human Genome Oligo Microarray slides 8×60K, washed, and scanned on an Agilent MicroArray Scanner (Agilent Technologies, Santa Clara, CA). Images were processed using Agilent Feature Extraction software (version 10.7.3.1). Gene expression differences are expressed relative to the respective spheroid DMSO control samples at the same time point. Microarray data were uploaded to the Gene Expression Omnibus database (submission number GSE93840).

#### Data Analysis.

Expression data were analyzed in Qlucore Omics Explorer 3.1 (Qlucore, Lund, Sweden). Gene set enrichment analyses were performed using WebGestalt (Wang et al., 2013). To assess statistical significances, heteroscedastic, two-tailed, unpaired *t* tests were performed and *P* values below 0.05 were considered significant. To correct for multiple tests, the Benjamini–Hochberg algorithm was used with false discovery rates (FDRs) as indicated.

## Results

### 

#### Transcriptional Characterization of Hepatic Cell Models.

**Fig. 1.**
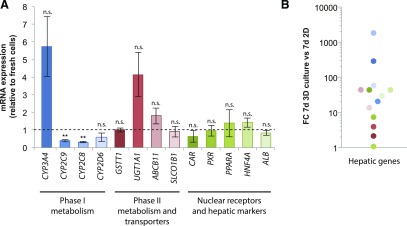
PHHs cultured in 3D spheroids resemble freshly isolated cells regarding expression patterns of drug-metabolizing enzymes, drug transporters, and hepatic markers. (A) Expression of phase I (*CYP2C8*, *CYP2C9*, *CYP2D6*, and *CYP3A4*) and phase II (*GSTT1* and *UGTA1*) metabolic enzymes, drug transporters (*SLCO1B1* and *ABCB11*), ligand-activated nuclear receptors (*CAR*, *PXR*, and *PPARA*) as well as the critical hepatic transcription factor *HNF4A* and the main hepatocyte secretory product, albumin (*ALB*), were quantified in PHH spheroids by quantitative polymerase chain reaction and normalized to expression in freshly isolated cells of the same donors (*n* = 3 to 4 donors; donor demographics are shown in Table 1). Importantly, with the exception of *CYP2C8* (33% of expression of freshly isolated cells, *P* = 0.001) and *CYP2C9* (40%, *P* = 0.004), no significant differences in expression levels between freshly isolated cells and PHH spheroids were detected. Error bars indicate S.E.M. **P < 0.01 (heteroscedastic two-tailed *t* test). (B) Expression levels of genes analyzed in (A) were elevated up to 1834-fold in the 3D spheroids compared with 2D cultured PHHs from the same donors after 7 days in culture. FC, Fold change; n.s., not significant (*P* > 0.05).

When cultured in 2D monolayers, PHHs rapidly dedifferentiate within hours, at least in part due to wide-scale microRNA-mediated inhibition of drug-metabolizing enzymes, transporters, and other hepatic genes (Elaut et al., 2006; Lauschke et al., 2016b,c). In contrast, expression levels of most important phase I (*CYP2C8*, *CYP2C9*, *CYP3A4*, and *CYP2D6*) and phase II (*GSTT1* and *UGT1A1*) drug-metabolizing enzymes, drug and bile transporters (*ABCB11* and *ABCC1* and *SLCO1B1*), ligand-activated nuclear receptors (*CAR*, *PXR*, and *PPARA*), and other genes with importance for hepatic functions (*ALB* and *HNF4A*) were preserved in 3D PHH spheroid cultures, approximating levels found in the corresponding freshly isolated cells (Fig. 1A). When hepatocytes from the same donors were cultured in 2D monolayers, expression of the same genes was downregulated up to 1800-fold, directly demonstrating the drastic effect of dedifferentiation on hepatic gene expression (Fig. 1B).

**Fig. 2.**
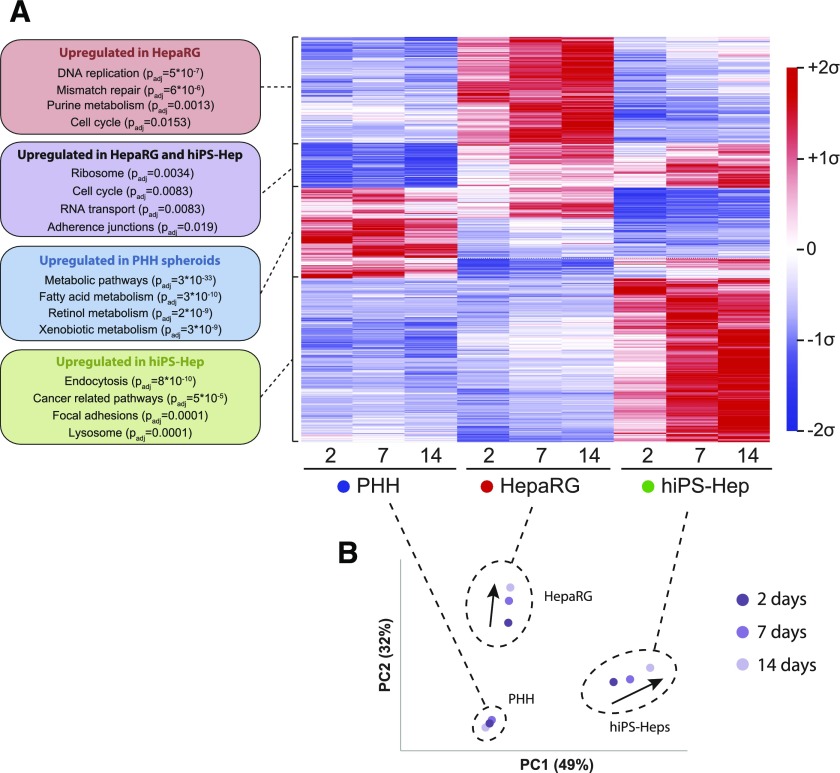
Transcriptomic profiling of hepatic in vitro models reveals wide-scale differences in global gene expression. (A) Heat map depicting differentially expressed genes in PHH spheroids (donor 1; blue), HepaRG cells (red), and hiPS-Hep cells (green) at 2, 7, and 14 days. Overall, 8148 of 17,462 genes analyzed were found to be differentially expressed after multiple testing correction (Benjamini–Hochberg FDR < 0.05). PHH spheroids showed elevated expression of genes involved in endogenous and xenobiotic metabolism (*P*_adjusted_ = 3 × 10^−33^), whereas HepaRG and hiPS-Hep cells exhibited, among others, elevated transcript levels of genes involved in proliferation (*P*_adjusted_ = 0.0083) and ribosomes (*P*_adjusted_ = 0.0034). Average values of three technical triplicates are presented as mean centered and *σ* normalized. (B) Principal component analysis revealed clear separation of the three cell models, which even increased over time (time progression is indicated as shades of purple). Notably, temporal changes of the transcriptomic signatures were more evident for HepaRG and hiPS-Hep cells during the culture period (indicated by arrows), whereas the transcriptomes of PHH spheroids remained temporally stable. PC, principal component.

We then benchmarked the mRNA expression patterns of PHH spheroids and HepaRG and hiPS-Hep cell systems using transcriptomic analyses (Fig. 2). Importantly, we found pronounced gene expression differences between the three models, with 8148 of 17,462 genes (47%) being differentially expressed over the course of 3 weeks in culture (Fig. 2A; FDR < 0.05, Benjamini–Hochberg correction). Genes involved in DNA replication (*P*_adjusted_ = 5 × 10^−7^), mismatch repair (*P*_adjusted_ = 6 × 10^−6^), and purine metabolism (*P*_adjusted_ = 0.0013) were significantly upregulated in HepaRG cells, whereas genes implicated in endocytosis (*P*_adjusted_ = 8 × 10^−10^), focal adhesion signaling (*P*_adjusted_ = 0.0001), and lysosomes (*P*_adjusted_ = 0.0001) were overexpressed in hiPS-Hep cells. In addition, pathways with general importance for cellular functions, such as ribosomes (*P*_adjusted_ = 0.0034), cell cycle (*P*_adjusted_ = 0.0083), and RNA transport (*P*_adjusted_ = 0.0083) were upregulated in both HepaRG and hiPS-Hep cells. Importantly, genes involved in the metabolism of endogenous as well as xenobiotic compounds were expressed at significantly elevated levels in PHH spheroids compared with HepaRG and hiPS-Hep cells (*P*_adjusted_ = 3 × 10^−33^). Although principal component analyses revealed pronounced changes over culture time in HepaRG and hiPS-Hep cells, gene expression signatures in PHH spheroids were stable over the course of 2 weeks (Fig. 2B).

**Fig. 3.**
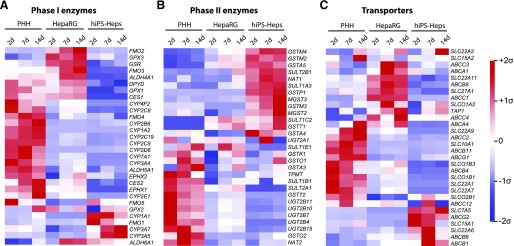
Expression levels of important ADME genes differ substantially between the three hepatic in vitro models. PHH spheroids, HepaRG cells, and hiPS-Hep cells showed pronounced expression differences in phase I enzymes (A), phase II enzymes (B), and drug transporters (C). Median expression values of three technical replicate microarray measurements are shown for each cell system and time point. Data are presented as mean centered and *σ* normalized. CES, Carboxylesterase; EPHX, Epoxide Hydrolase; FMO, Flavin Containing Monooxygenase; GPX, Glutathione Peroxidase; GSR, Glutathione-Disulfide Reductase; MGST, Microsomal Glutathione S-Transferase; NAT, N-Acetyltransferase; SULT, Sulfotransferase; TAP, ATP-Binding Cassette Transporter.

When focusing on genes with importance for drug ADME, we found that variations between the cell systems differed by gene class (Fig. 3). Levels of most phase I enzymes including major cytochrome P450 enzymes, such as *CYP1A2*, *CYP2B6*, *CYP2C8*, *CYP2C9*, and *CYP2D6*, were much higher in PHH spheroids compared with HepaRG and hiPS-Hep cells (Fig. 3A). *DPYD*, which encodes the rate-limiting enzyme in pyrimidine metabolism, was expressed at similar levels in PHH spheroids and HepaRG cells. In contrast, *CYP3A7* and *CYP3A5*, which constitute the major *CYP3A*s expressed in fetal liver (Hakkola et al., 2001), were highly expressed in hiPS-Hep cells.

Distinctly different sets of phase II enzymes were expressed in the three cell models. Expression of most transcripts encoding GST enzymes was highest in hiPS-Hep cells, and levels of *UGTs* and *TPMT* were elevated in 3D-cultured PHHs (Fig. 3B). Notably, phase II gene expression was generally low in HepaRG cells, suggesting a lower capacity of this cell model to accurately reflect and predict complex drug ADME patterns. Although relevant transporter genes were expressed in all three cell models, their relative abundances differed drastically (Fig. 3C). In PHH spheroids, high levels of physiologically important transporters—such as the bile acid transporters bile salt export pump (BSEP) and Na^+^-taurocholate cotransporting polypeptide (NTCP) encoded by *ABCB11* and *SLC10A1*, respectively; steroid and thyroid hormone transporters (*SLCO1B1* and *SLCO1B3*); and MDR2/3, the phosphatidylcholine transporter encoded by *ABCB4*—were observed. In contrast, transporters implicated in drug resistance of cancer cells were upregulated in hiPS-Hep cells, including *ABCB1* (MDR1) and *ABCG2* (BCRP) (Takara et al., 2006; Natarajan et al., 2012).

#### Toxicity in Hepatic Cell Systems under Repeated-Exposure Regimes.

**Fig. 4.**
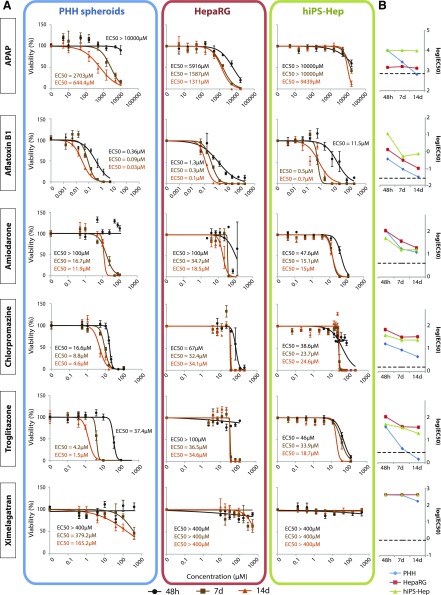
The sensitivity to model DILI compounds differs drastically between hiPS-Hep, HepaRG, and PHH cell models. (A) PHH spheroids, HepaRG cells, and hiPS-Hep cells were treated with APAP, aflatoxin B1, amiodarone, chlorpromazine, troglitazone, and ximelagatran in single-dose (48 hours, black) or repeated-exposure (7 days, brown; and 14 days, orange) experiments. Data are presented as the percentage relative to the viability of vehicle-treated controls at the same time point. For PHHs, two replicate experiments (both from donor 1) with six replicate measurements per concentration and time point are shown. For HepaRG cells, three replicate experiments with three replicate measurements per concentration and time point are shown. For hiPS-Hep cells, two replicate experiments with three replicate measurements per concentration and time point are shown. Error bars indicate S.E.M. (B) Semi-log plot showing the temporal evolution of sensitivity in PHHs (blue), HepaRG cells (red), and hiPS-Hep cells (green). Dashed lines indicate therapeutic exposure levels (human cmax). Note that long-term exposure resulted in increased sensitivity toward the hepatotoxins used in all cell systems. PHH spheroids detected toxicity at clinically relevant exposure levels for all compounds, with the exception of ximelagatran.

**Fig. 5.**
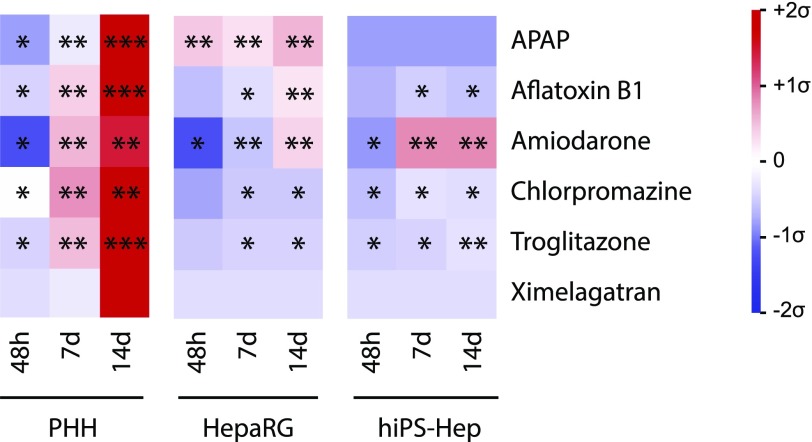
PHH spheroids constitute the most sensitive in vitro cell culture system tested. Heat map summarizing the sensitivities of the three cell systems to cytotoxicity, as shown in Fig. 4. Data are presented as mean centered and *σ* normalized and are related to therapeutic (ximelagatran and troglitazone) or toxic (APAP, aflatoxin B1, amiodarone, chlorpromazine) exposure values. Single, double, and triple asterisks indicate sensitivity < 30× *C*_max_, < 10× *C*_max_, and < 1× *C*_max_, respectively. *C*_max_ or exposure values were obtained from the following references: APAP, 700 *µ*M (Vale and Proudfoot, 1995); aflatoxin B1, 0.03 *µ*M (Hassan et al., 2006); amiodarone, 3.9 *µ*M (Regenthal et al., 1999); chlorpromazine, 1.6 *µ*M, (Regenthal et al., 1999); troglitazone, 2.82 *µ*M (Loi et al., 1999); and ximelagatran, 0.3 *µ*M, (Schützer et al., 2004).

Next, we investigated functional consequences of the observed expression differences. Previous studies have indicated that although PHHs provide a more predictive model than other hepatic cell lines, their predictive power in acute single-exposure studies in 2D cultures is significantly limited, at least in part due to the rapid loss of hepatic gene expression (Gerets et al., 2012; Sison-Young et al., 2016). Furthermore, with respect to the clinical profile of in vivo toxicity events, assessment of chronic drug-induced hepatotoxicity is of particular importance. Thus, here we investigated the effect of repeated-exposure regimens and analyzed the sensitivity of the three cell models to six hepatotoxic compounds that cause toxicity by distinctly different mechanisms (Figs. 4 and 5). We focused on 1) acetaminophen (APAP), which primarily causes hepatotoxicity due to reactive metabolite formation; 2) aflatoxin B1 as a genotoxic agent; 3) the antiarrhythmic drug amiodarone, which inhibits acyl-CoA transport and mitochondrial respiration; 4) the cholestatic agent chlorpromazine; 5) troglitazone as an inhibitor of *β*-oxidation that also causes direct opening of the mitochondrial permeability transition pore; and 6) the anticoagulant ximelagatran as a respiratory chain inhibitor.

The three cell systems showed drastic differences in their sensitivity to APAP toxicity. hiPS-Hep cells were insensitive to APAP toxicity, even after 14 days of treatment (14-day IC_50_ = 9439 *µ*M). In contrast, the HepaRG cell line detected toxicity already in the acute setting at high concentrations (48-hour IC_50_ = 5916 *µ*M) and the sensitivity increased further upon repeated exposures to approximate plasma levels in patients after acute APAP overdose (14-day IC_50_ = 1311 *µ*M; APAP plasma concentration for which immediate treatment is stipulated: >0.7–1.3 mM depending on additional risk factors; Vale and Proudfoot, 1995). In PHH spheroids, a drastic increase in sensitivity to APAP toxicity was apparent with chronic exposures, indicating toxicity slightly below typical overdose concentrations after 14 days of exposure (14-day IC_50_ = 644 *µ*M; therapeutic *C*_max_ = 136 *µ*M; Sevilla-Tirado et al., 2003).

Aflatoxin B1 toxicity showed substantial increases in toxicity over time in all cell systems. PHH spheroids were the most sensitive system in the acute as well as chronic setting, indicating toxicity at exposure levels detected in exposed individuals (28.5 nM; Hassan et al., 2006), followed by HepaRG cells.

hiPS-Hep cells were the only system to indicate amiodarone toxicity already after 48 hours, albeit only at high concentrations. After chronic exposure, all three cell models detected amiodarone-induced hepatotoxicity at similar concentrations, with PHH spheroids being the most sensitive, approximating exposure levels reported as toxic in patients (14-day IC_50_ = 11.9 *µ*M for PHHs, 18.5 *µ*M for HepaRG cells, and 15 *µ*M for hiPS-Hep cells; human toxic *C*_max_ = 3.8 *µ*M; Regenthal et al., 1999).

Although chlorpromazine-induced hepatic injury was detected by all three models, PHH spheroids were the most sensitive at all time points investigated and at concentrations approaching clinical exposure levels (14-day IC_50_ = 4.6 *µ*M for PHHs, 34.1 *µ*M for HepaRG cells, and 24.6 *µ*M for hiPS-Hep cells; human toxic *C*_max_ = 1.6 *µ*M; Regenthal et al., 1999).

Similarly, all three cell systems indicated troglitazone toxicity at clinically relevant concentrations, with IC_50_ values in PHH spheroids reaching therapeutic levels after chronic exposures (14-day IC_50_ = 1.5 *µ*M for PHHs, 34.6 *µ*M for HepaRG cells, and 18.7 *µ*M for hiPS-Hep cells; therapeutic *C*_max_ = 2.82 *µ*M; Loi et al., 1999).

PHH spheroids were the only system to indicate toxicity of ximelagatran after prolonged treatment (7 and 14 days), but only at relatively high concentrations that significantly exceeded therapeutic levels (14-day IC_50_ = 165 *µ*M; therapeutic *C*_max_ = 0.3 *µ*M; Schützer et al., 2004). It has previously proven difficult to detect ximelagatran toxicity in various in vitro systems (Kenne et al., 2008) and the mechanisms underlying this toxicity are still unclear, although evidence that ximelagatran inhibits mitochondrial respiration was recently presented (Neve et al., 2015).

In summary, although sensitivities differed between cell models for the hepatotoxic model compounds in the acute, single-dose setting, the PHH spheroid system was the most sensitive cell model after long-term exposure to all compounds tested (Fig. 5).

#### Toxicogenomic Analysis of Gene Expression Changes Preceding Compound Toxicity.

Next, we examined whether relevant compound-specific toxicity mechanisms were reflected using toxicogenomic profiling. To this end, we focused on the PHH spheroid model as the most sensitive system that detected toxicity of most tested compounds at clinically relevant exposure levels. To uncouple toxicity mechanisms and outcomes (i.e., study the changes in transcriptional signatures that precede the induction of cell death), we chose subtoxic concentrations (IC_10_) of the six model compounds. After 14 days of treatment, no significant expression changes were observed in APAP-, troglitazone-, and ximelagatran-treated samples (data not shown), suggesting that these compounds trigger cell death directly without extensive transcriptional perturbations.

**Fig. 6.**
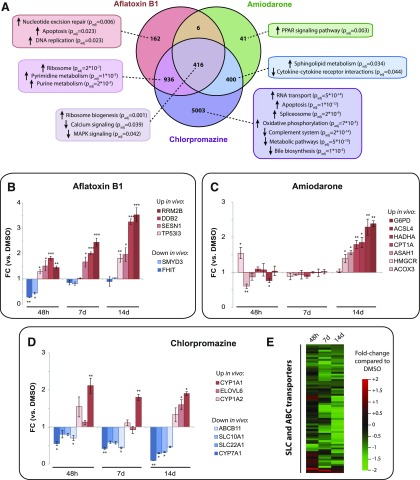
PHH spheroids faithfully mimic compound-specific transcriptional toxicity effects observed in vivo. Transcriptomic analyses of PHH spheroids treated chronically (14 days) with subtoxic concentrations (IC_10_) of aflatoxin B1, amiodarone, and chlorpromazine. (A) Venn diagram showing significantly dysregulated genes compared with DMSO controls (Benjamini–Hochberg multiple testing correction, FDR < 0.05). Gene set enrichment analysis revealed that compound-specific toxicity responses (e.g., DNA damage-related pathways, perturbations of bile acid metabolism, and PPAR signaling) were detected in aflatoxin B1–, chlorpromazine-, and amiodarone-treated spheroids. (B–D) Targeted analysis of genes implicated in aflatoxin B1 (B), amiodarone (C), and chlorpromazine (D) toxicity in vivo. Genes whose expression was up- or downregulated in vivo are shown in shades of red and blue, respectively. (E) Expression of cellular ABC and SLC transporters was broadly inhibited upon chlorpromazine treatment. *P < 0.05; **P < 0.01; ***P < 0.001 (heteroscedastic two-tailed *t* test compared with DMSO control at the same time point). ELOVL = Elongation Of Very Long Chain Fatty Acids Protein, FC = Fold change.

In contrast, pronounced changes of gene expression signatures were evident upon treatment with aflatoxin B1, amiodarone, and chlorpromazine (Fig. 6). Aflatoxin B1 induced nucleotide excision repair, apoptosis, and DNA replication (Fig. 6A), in agreement with its genotoxicity and with previous in vivo findings in aflatoxin-exposed rats and tree shrews (Ellinger-Ziegelbauer et al., 2004; Li et al., 2004; Jossé et al., 2012). We detected significant downregulation of *FHIT*, a tumor suppressor repressing canonical Wnt signaling by inhibition of *β*-catenin, whose activity is commonly impaired in preneoplastic lesions (Weiske et al., 2007). Similarly, we detected a reduction in levels of the methyltransferase *SMYD3*, which is implicated in hepatocellular carcinoma (Hamamoto et al., 2004) (Fig. 6B). Moreover, p53 signaling target genes, such as the p53 effector *TP53I3*, *RRM2B*, and *DDB2* (which play roles in DNA damage repair) and *SENS1* (a protein mediating the tumor-suppressive effect of p53 by inhibiting mechanistic target of rapamycin), were increasingly upregulated with prolonged exposure.

PPAR signaling was significantly upregulated after chronic amiodarone exposure, mimicking in vivo gene expression modulations in mice (McCarthy et al., 2004) (Fig. 6A), resulting in increased expression of e.g. *CPT1A*, a PPAR*α* target gene whose gene product is inhibited by amiodarone (Kennedy et al., 1996). Furthermore, we detected a progressive upregulation of key genes involved in lipid and cholesterol metabolism, such as *HADHA*, *ACSL4*, and *HMGCR* (Fig. 6C). Moreover, expression levels of *G6PD*, the central regulator of the pentose phosphate pathway that controls generation of NADPH, were significantly increased.

Prolonged chlorpromazine treatment caused the most pronounced perturbations of expression signatures, with 6755 genes identified as being differentially expressed (compared with 1520 for aflatoxin B1 and 863 for amiodarone). Among the deregulated pathways were bile acid metabolism (*P*_adjusted_ = 1 × 10^−5^), reflecting the cholestatic mechanism of chlorpromazine toxicity (Horikawa et al., 2003). Higher expression of *CYP1A2*, whose gene product is involved in chlorpromazine metabolism (Yoshii et al., 2000), increased with chlorpromazine treatment, whereas transcript levels of *CYP7A1*, the key enzyme in bile acid synthesis, as well as of the bile transporters *SLC22A1* (OCT1) and *SLC10A1* (NTCP) decreased. Moreover, expression levels of SLC and ABC transporters were broadly repressed after 14 days of treatment (Fig. 6E), suggesting major alterations of underlying transcriptional networks.

## Discussion

In this study, we compared the phenotypes of three emerging cell culture models for preclinical safety assessments of drugs and drug candidates: PHH spheroids, HepaRG cells, and hiPS-Hep cells. We found that mRNA expression levels of genes with importance for hepatic functionality in PHH spheroids pivoted around levels found in freshly isolated hepatocytes. These data corroborate the results of previous studies showing that 3D spheroid culture conditions improve the gene expression signatures and phenotypes of PHHs, resulting in an approximation of their physiologic counterparts in vivo in humans (Tostões et al., 2012; Bell et al., 2016). Importantly, transcriptional signatures of HepaRG and hiPS-Hep cells drastically differed, with 8148 of 17,462 genes (47% of the assessed transcriptome) being differentially expressed between the three cell models (FDR < 0.05). Importantly, expression of genes encoding enzymes involved in xenobiotic metabolism was strongly reduced in HepaRG and hiPS-Hep cells compared with PHH spheroids (*P*_adjusted_ = 3 × 10^−9^). Furthermore, HepaRG and hiPS-Hep cells exhibited impaired expression of genes involved in the metabolism of endogenous compounds, such as fatty acids (*P*_adjusted_ = 3 × 10^−10^) and retinol (*P*_adjusted_ = 2 × 10^−9^). Combined, these differences suggest impaired capacities of these two cell models to metabolize drugs and to faithfully mimic the mechanisms underlying compound toxicity.

When focusing on ADME genes, we detected highly elevated transcript levels of genes characteristic of the mature human liver, such as *CYP1A2*, *CYP2C8*, *CYP3A4*, *ABCB11*, and *SLC10A1*, in PHH spheroids. In contrast, hiPS-Hep cells showed increased levels of the fetal cytochrome P450s *CYP3A5* and *CYP3A7*, as well as high expression of the most important fetal GST (*GSTP1*) and transporters whose expression correlated with dedifferentiation during carcinogenesis, such as *ABCB1* and *ABCG2* (Hakkola et al., 2001; Raijmakers et al., 2001; Takara et al., 2006; Natarajan et al., 2012). The data revealed that gene expression signatures in PHH spheroids closely resembled those detected in isolated hepatocytes. In contrast, reduced expression of many important hepatic genes was evident in HepaRG and hiPS-Hep cells, indicative of deficits in maturation.

To relate changes in transcription patterns to functional consequences, we examined the differential sensitivities of the three cell models to hepatotoxins. APAP toxicity is primarily due to reactive metabolite formation catalyzed by CYP2E1 and CYP3A4 causing subsequent glutathione depletion, but immune-mediated mechanisms have also been linked to APAP-induced liver injury (reviewed in Krenkel et al., 2014). In agreement with high *CYP2E1* and *CYP3A4* expression levels and physiologic but comparatively low expression of GSTs involved in NAPQI detoxification, PHH spheroids detected APAP toxicity after 14 days at concentrations below typical overdose levels. The finding that APAP toxicity was already detected at concentrations that are clinically considered safe (Bradley et al., 1991; Geba et al., 2002) is consistent with previous clinical reports showing liver damage, as indicated by serum alanine aminotransferase elevations above three times the upper limit, in 31%–44% of healthy volunteers receiving 4 g APAP daily for 14 days (peak APAP serum level average = 99.2 *µ*M) (Watkins et al., 2006).

Similarly, hepatotoxicity of the mycotoxin aflatoxin B1 requires metabolic activation by CYP1A2 and CYP3A4 to a highly reactive 8,9-epoxide, which can lead to the development of hepatocellular carcinoma or, in rare cases, acute hepatotoxicity (Johnson and Guengerich, 1997; Macé et al., 1997; Williams et al., 2004). Sensitivity to aflatoxin B1 toxicity was strongly pronounced in PHH spheroids, which show physiologic expression levels of the respective metabolizing enzymes (Fig. 3A). Combined, these data suggest that physiologic and temporally stable expression levels of ADME genes are required to detect hepatotoxicity of compounds that require metabolic activation.

The lipophilic benzofuran derivative amiodarone causes mitochondrial uncoupling due to influx of protonated amiodarone into the mitochondrial matrix (Fromenty et al., 1990). Furthermore, it impairs the respiratory chain complexes I, II, and III and inhibits carnitine palmitoyltransferase I, thus limiting the import of fatty acids into the mitochondria and reducing the flux through mitochondrial *β*-oxidation (Fromenty et al., 1990; Kennedy et al., 1996; Spaniol et al., 2001). Sensitivity to amiodarone hepatotoxicity did not drastically increase over time and was similar between the three cell models. Although amiodarone is extensively metabolized by CYP3A4 and CYP2C8, its therapeutic as well as toxicological effects seem to be caused by both the parent compound as well as its dealkylated metabolite (Trivier et al., 1993; Soyama et al., 2002). Consequently, amiodarone toxicity does not depend on bioactivation, which could provide an explanation for the similar sensitivity levels between the cell systems. These findings are in agreement with previous reports showing lipid accumulation in hiPS-Hep and HepaRG cells already after short-term amiodarone exposures (Anthérieu et al., 2011; Pradip et al., 2016).

Chlorpromazine causes primarily cholestatic liver injury and multiple toxicity mechanisms have been suggested, including perturbation of oxidative phosphorylation (Nadanaciva et al., 2007), inhibition of bile export (Horikawa et al., 2003), glutathione depletion (Xu et al., 2008), phospholipidosis due to inhibition of phospholipases (Anderson and Borlak, 2006), and hypersensitivity (Ayd, 1956). Clinicopathologically, chlorpromazine toxicity manifests in approximately 1 in 100 patients typically 1–5 weeks after starting treatment and presents as self-limited jaundice, often in combination with eosinophilia (Selim and Kaplowitz, 1999, García Rodríguez et al., 1997). Most patients recover within weeks after discontinuation of treatment but few can experience progression of cholestatic injury to hepatic ductopenia. Toxicity of chlorpromazine was reported to be caused by its 7-hydroxylated metabolite, whereas the sulfoxidized metabolite appeared less toxic (Ros et al., 1979; Watson et al., 1988). PHH spheroids exhibited the highest sensitivity toward chlorpromazine and detected toxicity already at therapeutic concentrations, which was paralleled by increased expression of *CYP1A1* and *CYP1A2*, as previously reported (Parmentier et al., 2013). Furthermore, expression of genes with importance for bile acid synthesis [e.g., *CYP7A1*, which catalyzes the rate-limiting step in the classic bile acid synthesis pathway] and bile transport [e.g., the canalicular transporter BSEP (encoded by *ABCB11*) and the sinusoidal transporters NTCP (*SLC10A1*) and OCT1 (*SLC22A1*)] were strongly downregulated, mirroring expression alterations seen in patients with cholestasis in vivo (Zollner et al., 2001, 2007; Chen et al., 2008; Nies et al., 2009). Interestingly, transcriptional changes indicative of chlorpromazine-induced cholestasis preceded cytotoxicity by 2 weeks, suggesting the potential of the spheroid system to aid biomarker discovery.

The thiazolidinedione troglitazone is a PPAR*γ* agonist used as an insulin sensitizer for treatment of diabetes that also exhibits weak affinity to PPAR*α* (Lehmann et al., 1995). Troglitazone received regulatory approval in 1997 but was withdrawn from the US market in 2000 due to idiosyncratic hepatotoxicity. Troglitazone causes parent compound–mediated steatosis by inhibition of long-chain acyl-CoA synthetase and opening of the mitochondrial permeability transition pore (Fulgencio et al., 1996; Tirmenstein et al., 2002; Lim et al., 2008). In addition to parent compound toxicity, troglitazone metabolites, primarily troglitazone sulfate, can cause cholestatic liver injury by inhibition of BSEP, with an IC_50_ of 0.4 *µ*M (Funk et al., 2001). Furthermore, reactive metabolites and oxidative stress have been implicated in troglitazone toxicity, although their role remains controversial (comprehensively discussed in Masubuchi, 2006). The high sensitivity across models is consistent with troglitazone toxicity being largely caused by the parent compound itself. Nevertheless, toxicity in PHH spheroids is enhanced compared with the other two models, potentially due to additive effects of toxic metabolites such as troglitazone sulfate.

Notably, previous studies have demonstrated improved phenotypes, functionality, and sensitivity to various hepatotoxins in spheroid culture systems of hepatic cell lines (Fey and Wrzesinski, 2012; Gunness et al., 2013; Ramaiahgari et al., 2014), stem cell–derived HLCs (Takayama et al., 2013; Tasnim et al., 2016), and primary hepatocytes from rats (Sakai et al., 2010; Schutte et al., 2011; Purcell et al., 2014) and humans (Tostões et al., 2012; Bell et al., 2016). Yet toxicity in most of these studies was only tested under short-term exposure and was only detected at elevated concentrations (Table 2). Furthermore, whether the mechanisms of compound toxicity were recapitulated in vitro was not evaluated. Our study reinforces the positive effects of 3D culture on expression levels of hepatic genes and provides evidence that spheroids from PHHs can recapitulate human in vivo toxicity mechanisms in an in vitro setting.

**TABLE 2:** Comparison of the sensitivity to compounds tested in this study with published spheroid models

Cell Type	PHH		HepaRG		HepG2		HLC	C3A	Rat Hepatocytes
	Acute	Repeated Dose (14 Days)	Acute*^a^*,*^b^*	Repeated Dose (72 h)*^b^*	Acute*^b^**^,^**^c^**^,^**^d^*	Repeated Dose (6 Days)*^c^*	Acute*^d^**^,^**^e^*	Acute*^f^*	Acute*^g^*
APAP, mM	>10	0.6	3	ND	24	9.4	30	40	40
AFB, *µ*M	0.4	0.03	1.6	ND	>200	ND	7	ND	ND
AMD, *µ*M	>100	11.9	178	ND	50–>200	ND	25	260	ND
CPZ, *µ*M	16.6	4.6	98	43	43	ND	ND	ND	ND
TRO, *µ*M	37.4	1.5	400	ND	80–150	100	50	ND	ND
XIM, *µ*M	>400	165	ND	ND	ND	ND	ND	ND	ND

AFB, aflatoxin B1; AMD, amiodarone; CPZ, chlorpromazine; HLC, Stem cell-derived hepatocyte-like cells; ND, not determined; TRO, troglitazone; XIM, ximelagatran.

^*a*^ Gunness et al. (2013).

^*b*^ Mueller et al. (2014).

^*c*^ Ramaiahgari et al. (2014).

^*d*^ Takayama et al. (2013).

^*e*^ Tasnim et al. (2016).

^*f*^ Fey and Wrzesinski (2012).

^*g*^ Schutte et al. (2011).

Combined, the data presented here suggest that cytotoxicity studies in which long-term treatment regimens are employed improve the sensitivity of diverse hepatic in vitro models. PHH spheroids in particular were found to be the model that most accurately reflected in vivo expression signatures in the human liver. Consequently, 3D cultured PHHs were the most sensitive system to detect drug hepatotoxicity at clinically relevant concentrations. Furthermore, our results show that the 3D spheroid system faithfully reproduced transcriptional toxicity responses observed in human livers in vivo, particularly for drugs that require metabolic activation, act via reactive oxygen species, or inhibit bile flow. Thus, development and characterization of the 3D PHH spheroid model constitutes a promising step toward a much-needed physiologically replicative system that is mechanistically predictive of human drug response.

## Abbreviations

2D: two-dimensional
3D: three-dimensional
ABC: ATP-binding cassette
ADME: absorption, distribution, metabolism, and excretion
APAP: acetaminophen
BSEP: bile salt export pump
DILI: drug-induced liver injury
DMSO: dimethylsulfoxide
FBS: fetal bovine serum
FDR: false discovery rate
GST: glutathione-*S*-transferase
HLC: hepatocyte-like cell
iPSC: induced pluripotent stem cell
NAPQI: *N*-acetyl-*p*-benzoquinone imine
NTCP: Na^+^-taurocholate cotransporting polypeptide
PHH: primary human hepatocyte
PPAR: peroxisome proliferator-activated receptor
SLC: solute carrier organic anion

## References

[B1] Anderson N Borlak J (2006) Drug-induced phospholipidosis. FEBS Lett 580:5533–5540. 16979167 10.1016/j.febslet.2006.08.061

[B2] Anthérieu S Rogue A Fromenty B Guillouzo A Robin MA (2011) Induction of vesicular steatosis by amiodarone and tetracycline is associated with up-regulation of lipogenic genes in HepaRG cells. Hepatology 53:1895–1905. 21391224 10.1002/hep.24290

[B3] Ayd FJ Jr (1956) The dermatologic and systemic manifestations of chlorpromazine hypersensitivity; their clinical significance and management. J Nerv Ment Dis 124:84–87. 13416925 10.1097/00005053-195607000-00013

[B4] Bates DW Spell N Cullen DJ Burdick E Laird N Petersen LA Small SD Sweitzer BJ Leape LL Adverse Drug Events Prevention Study Group (1997) The costs of adverse drug events in hospitalized patients. JAMA 277:307–311. 9002493

[B5] Bell CC Hendriks DFG Moro SML Ellis E Walsh J Renblom A Fredriksson Puigvert L Dankers ACA Jacobs F Snoeys J (2016) Characterization of primary human hepatocyte spheroids as a model system for drug-induced liver injury, liver function and disease. Sci Rep 6:25187. 27143246 10.1038/srep25187 PMC4855186

[B6] Björnsson ES Bergmann OM Björnsson HK Kvaran RB Olafsson S (2013) Incidence, presentation, and outcomes in patients with drug-induced liver injury in the general population of Iceland. Gastroenterology 144:1419–1425. 23419359 10.1053/j.gastro.2013.02.006

[B7] Bradley JD Brandt KD Katz BP Kalasinski LA Ryan SI (1991) Comparison of an antiinflammatory dose of ibuprofen, an analgesic dose of ibuprofen, and acetaminophen in the treatment of patients with osteoarthritis of the knee. N Engl J Med 325:87–91. 2052056 10.1056/NEJM199107113250203

[B8] Chapman KL Holzgrefe H Black LE Brown M Chellman G Copeman C Couch J Creton S Gehen S Hoberman A (2013) Pharmaceutical toxicology: designing studies to reduce animal use, while maximizing human translation. Regul Toxicol Pharmacol 66:88–103. 23524271 10.1016/j.yrtph.2013.03.001

[B9] Chen HL Liu YJ Chen HL Wu SH Ni YH Ho MC Lai HS Hsu WM Hsu HY Tseng HC (2008) Expression of hepatocyte transporters and nuclear receptors in children with early and late-stage biliary atresia. Pediatr Res 63:667–673. 18327154 10.1203/PDR.0b013e318170a6b5

[B10] Classen DC Pestotnik SL Evans RS Lloyd JF Burke JP (1997) Adverse drug events in hospitalized patients. Excess length of stay, extra costs, and attributable mortality. JAMA 277:301–306. 9002492

[B11] Cook D Brown D Alexander R March R Morgan P Satterthwaite G Pangalos MN (2014) Lessons learned from the fate of AstraZeneca’s drug pipeline: a five-dimensional framework. Nat Rev Drug Discov 13:419–431. 24833294 10.1038/nrd4309

[B12] Elaut G Henkens T Papeleu P Snykers S Vinken M Vanhaecke T Rogiers V (2006) Molecular mechanisms underlying the dedifferentiation process of isolated hepatocytes and their cultures. Curr Drug Metab 7:629–660. 16918317 10.2174/138920006778017759

[B13] Ellinger-Ziegelbauer H Stuart B Wahle B Bomann W Ahr HJ (2004) Characteristic expression profiles induced by genotoxic carcinogens in rat liver. Toxicol Sci 77:19–34. 14600272 10.1093/toxsci/kfh016

[B14] Fey SJ Wrzesinski K (2012) Determination of drug toxicity using 3D spheroids constructed from an immortal human hepatocyte cell line. Toxicol Sci 127:403–411. 22454432 10.1093/toxsci/kfs122 PMC3355318

[B15] Fromenty B Fisch C Berson A Letteron P Larrey D Pessayre D (1990) Dual effect of amiodarone on mitochondrial respiration. Initial protonophoric uncoupling effect followed by inhibition of the respiratory chain at the levels of complex I and complex II. J Pharmacol Exp Ther 255:1377–1384. 1979817

[B16] Fulgencio JP Kohl C Girard J Pégorier JP (1996) Troglitazone inhibits fatty acid oxidation and esterification, and gluconeogenesis in isolated hepatocytes from starved rats. Diabetes 45:1556–1562. 8866561 10.2337/diab.45.11.1556

[B17] Funk C Pantze M Jehle L Ponelle C Scheuermann G Lazendic M Gasser R (2001) Troglitazone-induced intrahepatic cholestasis by an interference with the hepatobiliary export of bile acids in male and female rats. Correlation with the gender difference in troglitazone sulfate formation and the inhibition of the canalicular bile salt export pump (Bsep) by troglitazone and troglitazone sulfate. Toxicology 167:83–98. 11557132 10.1016/s0300-483x(01)00460-7

[B18] García Rodríguez LA Ruigómez A Jick H (1997) A review of epidemiologic research on drug-induced acute liver injury using the general practice research data base in the United Kingdom. Pharmacotherapy 17:721–728. 9250549

[B19] Geba GP Weaver AL Polis AB Dixon ME Schnitzer TJ Vioxx, Acetaminophen, Celecoxib Trial (VACT) Group (2002) Efficacy of rofecoxib, celecoxib, and acetaminophen in osteoarthritis of the knee: a randomized trial. JAMA 287:64–71. 11754710 10.1001/jama.287.1.64

[B20] Gerets HHJ Tilmant K Gerin B Chanteux H Depelchin BO Dhalluin S Atienzar FA (2012) Characterization of primary human hepatocytes, HepG2 cells, and HepaRG cells at the mRNA level and CYP activity in response to inducers and their predictivity for the detection of human hepatotoxins. Cell Biol Toxicol 28:69–87. 22258563 10.1007/s10565-011-9208-4 PMC3303072

[B21] Gómez-Lechón MJ Tolosa L Conde I Donato MT (2014) Competency of different cell models to predict human hepatotoxic drugs. Expert Opin Drug Metab Toxicol 10:1553–1568. 25297626 10.1517/17425255.2014.967680

[B22] Gunness P Mueller D Shevchenko V Heinzle E Ingelman-Sundberg M Noor F (2013) 3D organotypic cultures of human HepaRG cells: a tool for in vitro toxicity studies. Toxicol Sci 133:67–78. 23377618 10.1093/toxsci/kft021

[B23] Hakkola J Raunio H Purkunen R Saarikoski S Vähäkangas K Pelkonen O Edwards RJ Boobis AR Pasanen M (2001) Cytochrome P450 3A expression in the human fetal liver: evidence that CYP3A5 is expressed in only a limited number of fetal livers. Biol Neonate 80:193–201. 11585982 10.1159/000047142

[B24] Hamamoto R Furukawa Y Morita M Iimura Y Silva FP Li M Yagyu R Nakamura Y (2004) SMYD3 encodes a histone methyltransferase involved in the proliferation of cancer cells. Nat Cell Biol 6:731–740. 15235609 10.1038/ncb1151

[B25] Hassan AM Sheashaa HA Abdel Fatah MF Ibrahim AZ Gaber OA (2006) Does aflatoxin as an environmental mycotoxin adversely affect the renal and hepatic functions of Egyptian lactating mothers and their infants? A preliminary report. Int Urol Nephrol 38:339–342. 16868707 10.1007/s11255-006-0056-8

[B26] Heslop JA Rowe C Walsh J Sison-Young R Jenkins R Kamalian L Kia R Hay D Jones RP Malik HZ (2017) Mechanistic evaluation of primary human hepatocyte culture using global proteomic analysis reveals a selective dedifferentiation profile. Arch Toxicol 91:439–452. 27039104 10.1007/s00204-016-1694-y PMC5225178

[B27] Horikawa M Kato Y Tyson CA Sugiyama Y (2003) Potential cholestatic activity of various therapeutic agents assessed by bile canalicular membrane vesicles isolated from rats and humans. Drug Metab Pharmacokinet 18:16–22. 15618715 10.2133/dmpk.18.16

[B28] Johnson WW Guengerich FP (1997) Reaction of aflatoxin B1 exo-8,9-epoxide with DNA: kinetic analysis of covalent binding and DNA-induced hydrolysis. Proc Natl Acad Sci USA 94:6121–6125. 9177180 10.1073/pnas.94.12.6121 PMC21012

[B29] Jossé R Dumont J Fautrel A Robin MA Guillouzo A (2012) Identification of early target genes of aflatoxin B1 in human hepatocytes, inter-individual variability and comparison with other genotoxic compounds. Toxicol Appl Pharmacol 258:176–187. 22100608 10.1016/j.taap.2011.10.019

[B30] Kaplowitz N (2005) Idiosyncratic drug hepatotoxicity. Nat Rev Drug Discov 4:489–499. 15931258 10.1038/nrd1750

[B31] Kenne K Skanberg I Glinghammar B Berson A Pessayre D Flinois JP Beaune P Edebert I Pohl CD Carlsson S (2008) Prediction of drug-induced liver injury in humans by using in vitro methods: the case of ximelagatran. Toxicol In Vitro 22:730–746. 18191936 10.1016/j.tiv.2007.11.014

[B32] Kennedy JA Unger SA Horowitz JD (1996) Inhibition of carnitine palmitoyltransferase-1 in rat heart and liver by perhexiline and amiodarone. Biochem Pharmacol 52:273–280. 8694852 10.1016/0006-2952(96)00204-3

[B33] Kia R Sison RLC Heslop J Kitteringham NR Hanley N Mills JS Park BK Goldring CEP (2013) Stem cell-derived hepatocytes as a predictive model for drug-induced liver injury: are we there yet? Br J Clin Pharmacol 75:885–896. 22703588 10.1111/j.1365-2125.2012.04360.x PMC3612706

[B34] Klein S Mueller D Schevchenko V Noor F (2014) Long-term maintenance of HepaRG cells in serum-free conditions and application in a repeated dose study. J Appl Toxicol 34:1078–1086. 24114766 10.1002/jat.2929

[B35] Krenkel O Mossanen JC Tacke F (2014) Immune mechanisms in acetaminophen-induced acute liver failure. Hepatobiliary Surg Nutr 3:331–343. 25568858 10.3978/j.issn.2304-3881.2014.11.01 PMC4273118

[B36] Lasser KE Allen PD Woolhandler SJ Himmelstein DU Wolfe SM Bor DH (2002) Timing of new black box warnings and withdrawals for prescription medications. JAMA 287:2215–2220. 11980521 10.1001/jama.287.17.2215

[B37] Lauschke VM Hendriks DFG Bell CC Andersson TB Ingelman-Sundberg M (2016 a) Novel 3D culture systems for studies of human liver function and assessments of the hepatotoxicity of drugs and drug candidates. *Chem Res Toxicol* 29:1936–1955. 10.1021/acs.chemrestox.6b00150 27661221

[B38] Lauschke VM Ingelman-Sundberg M (2016) The importance of patient-specific factors for hepatic drug response and toxicity. Int J Mol Sci 17:1714–1727. 10.3390/ijms17101714 PMC5085745 27754327

[B39] Lauschke VM Mkrtchian S Ingelman-Sundberg M (2016 b) The role of microRNAs in liver injury at the crossroad between hepatic cell death and regeneration. Biochem Biophys Res Commun DOI: 10.1016/j.bbrc.2016.10.084 [published ahead of print]. 10.1016/j.bbrc.2016.10.084 27789285

[B40] Lauschke VM Vorrink SU Moro SM Rezayee F Nordling Å Hendriks DF Bell CC Sison-Young R Park BK Goldring CE (2016 c) Massive rearrangements of cellular microRNA signatures are key drivers of hepatocyte dedifferentiation. Hepatology 64:1743–1756. 27532775 10.1002/hep.28780

[B41] Lehmann JM Moore LB Smith-Oliver TA Wilkison WO Willson TM Kliewer SA (1995) An antidiabetic thiazolidinedione is a high affinity ligand for peroxisome proliferator-activated receptor gamma (PPAR gamma). J Biol Chem 270:12953–12956. 7768881 10.1074/jbc.270.22.12953

[B42] Li Y Wan DF Su JJ Cao J Ou C Qiu XK Ban KC Yang C Qin LL Luo D (2004) Differential expression of genes during aflatoxin B(1)-induced hepatocarcinogenesis in tree shrews. World J Gastroenterol 10:497–504. 14966905 10.3748/wjg.v10.i4.497 PMC4716968

[B43] Lim PLK Liu J Go ML Boelsterli UA (2008) The mitochondrial superoxide/thioredoxin-2/Ask1 signaling pathway is critically involved in troglitazone-induced cell injury to human hepatocytes. Toxicol Sci 101:341–349. 17975114 10.1093/toxsci/kfm273

[B44] Loi CM Alvey CW Vassos AB Randinitis EJ Sedman AJ Koup JR (1999) Steady-state pharmacokinetics and dose proportionality of troglitazone and its metabolites. J Clin Pharmacol 39:920–926. 10471982 10.1177/00912709922008533

[B45] Macé K Aguilar F Wang JS Vautravers P Gómez-Lechón M Gonzalez FJ Groopman J Harris CC Pfeifer AM (1997) Aflatoxin B1-induced DNA adduct formation and p53 mutations in CYP450-expressing human liver cell lines. Carcinogenesis 18:1291–1297. 9230270 10.1093/carcin/18.7.1291

[B46] Masubuchi Y (2006) Metabolic and non-metabolic factors determining troglitazone hepatotoxicity: a review. Drug Metab Pharmacokinet 21:347–356. 17072088 10.2133/dmpk.21.347

[B47] McCarthy TC Pollak PT Hanniman EA Sinal CJ (2004) Disruption of hepatic lipid homeostasis in mice after amiodarone treatment is associated with peroxisome proliferator-activated receptor-alpha target gene activation. J Pharmacol Exp Ther 311:864–873. 15265979 10.1124/jpet.104.072785

[B48] Mueller D, Krämer L, Hoffmann E, Klein S, and Noor F (2014) 3D organotypic HepaRG cultures as in vitro model for acute and repeated dose toxicity studies. *Toxicol In Vitro* 28:104–112. 10.1016/j.tiv.2013.06.024 23850736

[B49] Nadanaciva S Bernal A Aggeler R Capaldi R Will Y (2007) Target identification of drug induced mitochondrial toxicity using immunocapture based OXPHOS activity assays. Toxicol In Vitro 21:902–911. 17346924 10.1016/j.tiv.2007.01.011

[B50] Natarajan K Xie Y Baer MR Ross DD (2012) Role of breast cancer resistance protein (BCRP/ABCG2) in cancer drug resistance. Biochem Pharmacol 83:1084–1103. 22248732 10.1016/j.bcp.2012.01.002 PMC3307098

[B51] Neve EPA Köfeler H Hendriks DFG Nordling Å Gogvadze V Mkrtchian S Näslund E Ingelman-Sundberg M (2015) Expression and function of mARC: roles in lipogenesis and metabolic activation of ximelagatran. PLoS One 10:e0138487. 26378779 10.1371/journal.pone.0138487 PMC4574727

[B52] Nies AT Koepsell H Winter S Burk O Klein K Kerb R Zanger UM Keppler D Schwab M Schaeffeler E (2009) Expression of organic cation transporters OCT1 (SLC22A1) and OCT3 (SLC22A3) is affected by genetic factors and cholestasis in human liver. Hepatology 50:1227–1240. 19591196 10.1002/hep.23103

[B53] Ostapowicz G Fontana RJ Schiødt FV Larson A Davern TJ Han SHB McCashland TM Shakil AO Hay JE Hynan L U.S. Acute Liver Failure Study Group (2002) Results of a prospective study of acute liver failure at 17 tertiary care centers in the United States. Ann Intern Med 137:947–954. 12484709 10.7326/0003-4819-137-12-200212170-00007

[B54] Park BK Boobis A Clarke S Goldring CEP Jones D Kenna JG Lambert C Laverty HG Naisbitt DJ Nelson S (2011) Managing the challenge of chemically reactive metabolites in drug development. Nat Rev Drug Discov 10:292–306. 21455238 10.1038/nrd3408

[B55] Parmentier C Truisi GL Moenks K Stanzel S Lukas A Kopp-Schneider A Alexandre E Hewitt PG Mueller SO Richert L (2013) Transcriptomic hepatotoxicity signature of chlorpromazine after short- and long-term exposure in primary human sandwich cultures. Drug Metab Dispos 41:1835–1842. 23913027 10.1124/dmd.113.052415

[B56] Pradip A Steel D Jacobsson S Holmgren G Ingelman-Sundberg M Sartipy P Björquist P Johansson I Edsbagge J (2016) High content analysis of human pluripotent stem cell derived hepatocytes reveals drug induced steatosis and phospholipidosis. Stem Cells Int 2016:2475631. 26880940 10.1155/2016/2475631 PMC4736406

[B57] Purcell SM Moran JL Fromer M Ruderfer D Solovieff N Roussos P O’Dushlaine C Chambert K Bergen SE Kähler A (2014) A polygenic burden of rare disruptive mutations in schizophrenia. Nature 506:185–190. 24463508 10.1038/nature12975 PMC4136494

[B58] Raijmakers MT Steegers EA Peters WH (2001) Glutathione S-transferases and thiol concentrations in embryonic and early fetal tissues. Hum Reprod 16:2445–2450. 11679536 10.1093/humrep/16.11.2445

[B59] Ramaiahgari SC den Braver MW Herpers B Terpstra V Commandeur JNM van de Water B Price LS (2014) A 3D in vitro model of differentiated HepG2 cell spheroids with improved liver-like properties for repeated dose high-throughput toxicity studies. Arch Toxicol 88:1083–1095. 24599296 10.1007/s00204-014-1215-9

[B60] Regenthal R Krueger M Koeppel C Preiss R (1999) Drug levels: therapeutic and toxic serum/plasma concentrations of common drugs. J Clin Monit Comput 15:529–544. 12578052 10.1023/a:1009935116877

[B61] Ros E Small DM Carey MC (1979) Effects of chlorpromazine hydrochloride on bile salt synthesis, bile formation and biliary lipid secretion in the rhesus monkey: a model for chlorpromazine-induced cholestasis. Eur J Clin Invest 9:29–41. 110598 10.1111/j.1365-2362.1979.tb01664.x

[B62] Russo MW Galanko JA Shrestha R Fried MW Watkins P (2004) Liver transplantation for acute liver failure from drug induced liver injury in the United States. Liver Transpl 10:1018–1023. 15390328 10.1002/lt.20204

[B63] Sakai Y Yamagami S Nakazawa K (2010) Comparative analysis of gene expression in rat liver tissue and monolayer- and spheroid-cultured hepatocytes. Cells Tissues Organs 191:281–288. 20051666 10.1159/000272316

[B64] Schutte M Fox B Baradez M-O Devonshire A Minguez J Bokhari M Przyborski S Marshall D (2011) Rat primary hepatocytes show enhanced performance and sensitivity to acetaminophen during three-dimensional culture on a polystyrene scaffold designed for routine use. Assay Drug Dev Technol 9:475–486. 21675871 10.1089/adt.2011.0371

[B65] Schützer KM Wall U Lönnerstedt C Ohlsson L Teng R Sarich TC Eriksson UG (2004) Bioequivalence of ximelagatran, an oral direct thrombin inhibitor, as whole or crushed tablets or dissolved formulation. Curr Med Res Opin 20:325–331. 15025841 10.1185/030079903125003035

[B66] Selim K Kaplowitz N (1999) Hepatotoxicity of psychotropic drugs. Hepatology 29:1347–1351. 10216114 10.1002/hep.510290535

[B67] Sevilla-Tirado FJ González-Vallejo EB Leary AC Breedt HJ Hyde VJ Fernández-Hernando N (2003) Bioavailability of two new formulations of paracetamol, compared with three marketed formulations, in healthy volunteers. Methods Find Exp Clin Pharmacol 25:531–535. 14571283 10.1358/mf.2003.25.7.778092

[B68] Sgro C Clinard F Ouazir K Chanay H Allard C Guilleminet C Lenoir C Lemoine A Hillon P (2002) Incidence of drug-induced hepatic injuries: a French population-based study. Hepatology 36:451–455. 12143055 10.1053/jhep.2002.34857

[B69] Sison-Young RL Lauschke VM Johann E Alexandre E Anthérieu S Aerts H Gerets HHJ Labbe G Hoët D Dorau M (2016) A multicenter assessment of single-cell models aligned to standard measures of cell health for prediction of acute hepatotoxicity. Arch Toxicol DOI: 10.1007/s00204-016-1745-4 [published online ahead of print]. 10.1007/s00204-016-1745-4 PMC5316403 27344343

[B70] Soyama A Hanioka N Saito Y Murayama N Ando M Ozawa S Sawada J (2002) Amiodarone N‐deethylation by CYP2C8 and its variants, CYP2C8*3 and CYP2C8 P404A. Pharmacol Toxicol 91:174–178. 12530467 10.1034/j.1600-0773.2002.910404.x

[B71] Spaniol M Bracher R Ha HR Follath F Krähenbühl S (2001) Toxicity of amiodarone and amiodarone analogues on isolated rat liver mitochondria. J Hepatol 35:628–636. 11690709 10.1016/s0168-8278(01)00189-1

[B72] Takara K Sakaeda T Okumura K (2006) An update on overcoming MDR1-mediated multidrug resistance in cancer chemotherapy. Curr Pharm Des 12:273–286. 16454744 10.2174/138161206775201965

[B73] Takayama K Kawabata K Nagamoto Y Kishimoto K Tashiro K Sakurai F Tachibana M Kanda K Hayakawa T Furue MK (2013) 3D spheroid culture of hESC/hiPSC-derived hepatocyte-like cells for drug toxicity testing. Biomaterials 34:1781–1789. 23228427 10.1016/j.biomaterials.2012.11.029

[B74] Tasnim F Toh YC Qu Y Li H Phan D Narmada BC Ananthanarayanan A Mittal N Meng RQ Yu H (2016) Functionally enhanced human stem cell derived hepatocytes in galactosylated cellulosic sponges for hepatotoxicity testing. Mol Pharm 13:1947–1957. 27157693 10.1021/acs.molpharmaceut.6b00119

[B75] Tirmenstein MA Hu CX Gales TL Maleeff BE Narayanan PK Kurali E Hart TK Thomas HC Schwartz LW (2002) Effects of troglitazone on HepG2 viability and mitochondrial function. Toxicol Sci 69:131–138. 12215667 10.1093/toxsci/69.1.131

[B76] Tostões RM Leite SB Serra M Jensen J Björquist P Carrondo MJT Brito C Alves PM (2012) Human liver cell spheroids in extended perfusion bioreactor culture for repeated-dose drug testing. Hepatology 55:1227–1236. 22031499 10.1002/hep.24760

[B77] Trivier JM Libersa C Belloc C Lhermitte M (1993) Amiodarone N-deethylation in human liver microsomes: involvement of cytochrome P450 3A enzymes (first report). Life Sci 52:PL91–PL96. 8445979 10.1016/0024-3205(93)90523-6

[B78] Vale JA Proudfoot AT (1995) Paracetamol (acetaminophen) poisoning. Lancet 346:547–552. 7658783 10.1016/s0140-6736(95)91385-8

[B79] Wang J Duncan D Shi Z Zhang B (2013) WEB-based GEne SeT AnaLysis Toolkit (WebGestalt): update 2013. Nucleic Acids Res 41:W77–W83. 23703215 10.1093/nar/gkt439 PMC3692109

[B80] Watkins PB Kaplowitz N Slattery JT Colonese CR Colucci SV Stewart PW Harris SC (2006) Aminotransferase elevations in healthy adults receiving 4 grams of acetaminophen daily: a randomized controlled trial. JAMA 296:87–93. 16820551 10.1001/jama.296.1.87

[B81] Watson RG Olomu A Clements D Waring RH Mitchell S Elias E (1988) A proposed mechanism for chlorpromazine jaundice--defective hepatic sulphoxidation combined with rapid hydroxylation. J Hepatol 7:72–78. 3183354 10.1016/s0168-8278(88)80508-7

[B82] Weiske J Albring KF Huber O (2007) The tumor suppressor Fhit acts as a repressor of beta-catenin transcriptional activity. Proc Natl Acad Sci USA 104:20344–20349. 18077326 10.1073/pnas.0703664105 PMC2154433

[B83] Williams JH Phillips TD Jolly PE Stiles JK Jolly CM Aggarwal D (2004) Human aflatoxicosis in developing countries: a review of toxicology, exposure, potential health consequences, and interventions. Am J Clin Nutr 80:1106–1122. 15531656 10.1093/ajcn/80.5.1106

[B84] Xu JJ Henstock PV Dunn MC Smith AR Chabot JR de Graaf D (2008) Cellular imaging predictions of clinical drug-induced liver injury. Toxicol Sci 105:97–105. 18524759 10.1093/toxsci/kfn109

[B85] Yoshii K Kobayashi K Tsumuji M Tani M Shimada N Chiba K (2000) Identification of human cytochrome P450 isoforms involved in the 7-hydroxylation of chlorpromazine by human liver microsomes. Life Sci 67:175–184. 10901285 10.1016/s0024-3205(00)00613-5

[B86] Zollner G Fickert P Zenz R Fuchsbichler A Stumptner C Kenner L Ferenci P Stauber RE Krejs GJ Denk H (2001) Hepatobiliary transporter expression in percutaneous liver biopsies of patients with cholestatic liver diseases. Hepatology 33:633–646. 11230744 10.1053/jhep.2001.22646

[B87] Zollner G Wagner M Fickert P Silbert D Gumhold J Zatloukal K Denk H Trauner M (2007) Expression of bile acid synthesis and detoxification enzymes and the alternative bile acid efflux pump MRP4 in patients with primary biliary cirrhosis. Liver Int 27:920–929. 17696930 10.1111/j.1478-3231.2007.01506.x

